# Preventable proportion of intubation-associated pneumonia: Role of adherence to a care bundle

**DOI:** 10.1371/journal.pone.0181170

**Published:** 2017-09-06

**Authors:** Antonella Agodi, Martina Barchitta, Annalisa Quattrocchi, Emiliano Spera, Giovanni Gallo, Francesco Auxilia, Silvio Brusaferro, Marcello Mario D’Errico, Maria Teresa Montagna, Cesira Pasquarella, Stefano Tardivo, Ida Mura, on behalf of the GISIO-SItI working group

**Affiliations:** 1 Department of Medical and Surgical Sciences and Advanced Technologies “GF Ingrassia”, University of Catania, Catania, Italy; 2 Department of Mathematics and Informatics, University of Catania, Catania, Italy; 3 Department of Biomedical Sciences for Health, University of Milan, Milan, Italy; 4 Department of Medicine, University of Udine, Udine, Italy; 5 Department of Biomedical Sciences and Public Health, Polytechnic University of Marche, Ancona, Italy; 6 Department of Biomedical Sciences and Human Oncology, University of Bari ‘Aldo Moro’, Bari, Italy; 7 Department of Medicine and Surgery, University of Parma, Parma, Italy; 8 Department of Diagnostic and Public Health, University of Verona, Verona, Italy; 9 Department of Biomedical Sciences, University of Sassari, Sassari, Italy; 10 GISIO-SItI working group: Italian Study Group of Hospital Hygiene - Italian Society of Hygiene, Preventive Medicine and Public Health, Italy; National Yang-Ming University, TAIWAN

## Abstract

**Objective:**

The aim of the present study was to estimate the preventable proportion of Intubation-Associated Pneumonia (IAP) in the Intensive Care Units (ICUs) participating in the Italian Nosocomial Infections Surveillance in ICUs (SPIN-UTI) network, taking into account differences in intrinsic patients’ risk factors, and additionally considering the compliance with the European bundle for IAP prevention.

**Methods:**

A prospective patient-based survey was conducted and all patients staying in ICU for more than 2 days were enrolled in the surveillance. Compliance with the bundle was assessed using a questionnaire for each intubated patient. A twofold analysis by the parametric g-formula was used to compute the number of infections to be expected if the infection incidence in all ICUs could be reduced to that one of the top-tenth-percentile-ranked ICUs and to that one of the ICU with the highest compliance to all five bundle components.

**Results:**

A total of 1,840 patients and of 17 ICUs were included in the first analysis showing a preventable proportion of 44% of IAP. In a second analysis on a subset of data, considering compliance with the European bundle, a preventable proportion of 40% of IAP was shown. A significant negative trend of IAP incidences was observed with increasing number of bundle components performed (p<0.001) and a strong negative correlation between these two factors was shown (r = -0.882; p = 0.048).

**Conclusions:**

The g-formula controlled for time-varying factors is a valuable approach for estimating the preventable proportion of IAP and the impact of interventions, based entirely on an observed population in a real-world setting. However, both the study design that cannot definitively prove a causative relationship between bundle compliance and IAP risk, and the small number of patients included in the care bundle compliance analysis, may represent limits of the study and further and larger studies should be conducted.

## Introduction

Healthcare-Associated Infections (HAIs), and especially those acquired in Intensive Care Units (ICUs), comprise the largest part of adverse events in the healthcare setting and affect patient morbidity and mortality. HAIs cause prolonged ICU and hospital length of stay, excessive utilization of antimicrobials and elevated costs [1, 2].

Surveillance of device-associated infections especially in ICUs is gaining in importance [3, 4]. In Europe, in 2012, ICU-acquired pneumonia occurred in 5.3% of the patients staying more than 2 days in ICUs and 92% of these infections were associated with mechanical ventilation [5]. The Italian Nosocomial Infections Surveillance in Intensive Care Units (ICUs) (Sorveglianza Prospettica delle Infezioni Nosocomiali nelle Unità di Terapia Intensiva, SPIN-UTI), network reported that pneumonia occurred in 9.8% of patients admitted to the ICU and 96% of these infections were associated with intubation (Intubation-Associated Pneumonia, IAP). Furthermore, in the SPIN-UTI network an increasing trend of IAP rates was observed from 2006 to 2015 [6–10]. The burden and adverse effects of ICU-acquired pneumonia on healthcare outcomes have increased pressure on clinicians and healthcare systems to prevent this infection [11]. A recent European study has estimated that up to 52% of ventilator-associated pneumonia is preventable [12]. The implementation in clinical practice of care bundles for prevention of ventilator-associated pneumonia, as that developed by a pan-European committee [13], has been widely encouraged in intubated ICU-patients and is associated with a reduced risk of pneumonia [14].

In healthcare settings, given the variety of possible combinations of interventions, controlled studies are difficult to conduct. The aim of the present study was to estimate, using routinely collected data, the preventable proportion of IAP in the ICUs participating in the SPIN-UTI network, taking into account differences in intrinsic risk factors in their patient population, and additionally considering the degree of compliance with the European bundle for IAP prevention. Therefore, a twofold analysis was performed to estimate the expected number of IAP and the incidence that would be realized if all ICUs had the same incidence as ICUs with: (i) the lowest IAP incidence, and (ii) the highest compliance to all five components of the bundle.

To the best of our knowledge, the present study is the first that evaluated the efficacy of care bundle compliance for prevention of ventilator-associated pneumonia, in terms of preventable proportion of IAP, through a model-based simulation.

## Material and methods

### Surveillance study design

The SPIN-UTI network was established in Italy by the Italian Study Group of Hospital Hygiene (GISIO) of the Italian Society of Hygiene, Preventive Medicine and Public Health (SItI) [6, 7, 8]. Patient-based surveillance data used in the present analysis were collected during the fourth edition of the surveillance study, between 1^st^ October 2012 and 30^th^ June 2013, according to the European HAI-ICU protocol for patient-based surveillance [4]. Briefly, ICUs prospectively collected data for all patients staying for more than 2 days. Data related to hospital and ICU characteristics, to patients (i.e. ICU stay information, patient risk factors, device exposure, antimicrobial use and patient outcome), infection status (i.e. infection date, infection site and associated-microorganisms) and microorganisms (i.e. antimicrobial resistance data) were collected by infection control practitioners, intensive care specialists and other personnel trained in the surveillance methodology and in European HAI-ICU definitions. A web-based data collection procedure by means of four electronic data forms, designed using an on-line platform, was adopted. Presence of invasive devices (e. g. intubation and Central Venous Catheter, CVC) were recorded daily [6, 7, 8]. Pneumonia was defined according to standard case definitions reported in the HAI-ICU protocol (a combination of clinical, radiological, and microbiological criteria) and considered as IAP if an invasive respiratory device—by either tracheostomy or endotracheal tube—was present, even intermittently, in the 48 hours preceding the onset of infection [4].

A validation study was performed at the end of the first surveillance survey, from November 2006 to May 2007, in order to validate infection data reported on patients in the ICUs participating in the SPIN-UTI network: the overall sensitivity was 82.3% and the overall specificity was 97.2% [7].

The incidence of IAP was computed as the number of IAP divided by the number of intubated patients and the IAP rate as the number of IAP divided by the number of intubation-days.

This project was approved by the Italian Ministry of Health—CCM 2012. No consent from patients was necessary as the data were analyzed anonymously.

### Bundle compliance

In the framework of the surveillance study, between 1^st^ January and 30^th^ June 2013, in order to determine compliance with the European bundle components for prevention of ventilator-associated pneumonia [13, 14], on a subgroup of 15 ICUs that voluntarily agree to participate, an *ad hoc* questionnaire was filled out for each intubated patient, as previously reported [9, 10]. Particularly, for each intubated patient, during ICU stay, compliance with each of the five bundle components were checked and recorded by the surveillance personnel as dichotomous variables (yes/no). The five bundle components checked throughout the ICU stay were: (i) no ventilator circuit tube changes; (ii) daily sedation vacation and weaning protocol (i.e. daily sedation interruption and daily assessment of readiness to extubate); (iii) strict hand hygiene using alcohol-based antiseptic before manipulating the airways; (iv) oral care with chlorhexidine 0.12% every 8 h; and (v) intra-cuff pressure control to reduce leakage of oropharyngeal secretions to the lower airways tract [13].

Compliance with each bundle component was calculated as the number of intubated patients for whom a specific component of the bundle was documented and in place during ICU stay, over the total number of intubated patients observed in the same period of time.

The correlation between incidence of IAP (per 100 intubated patients) and the number of bundle components performed was assessed using linear regression and the Pearson correlation coefficient.

### Statistical methods

A detailed statistical report is available as Supplementary material (S1 Text). The model used in the present study was previously adopted using a large European cohort study database [12]. The parametric g-formula, a generalization of standardization used to adjust for time-varying confounders affected by prior exposures [15], was used for computing the preventable proportion of IAP. The aim of this analysis was to estimate the expected number of IAP and the incidence that would be realized if ICUs with high IAP incidence (referred to as “other ICUs”), had the same incidence as reference ICUs (those with the IAP incidences below or equal to the 10^th^ percentile of the distribution, (referred to as “best ICUs”) after adjusting for patients and ICU characteristics. From the original SPIN-UTI database, a day-by-day database was generated considering device exposure, IAP and bloodstream infection (BSI) status, for each patient and for each day of ICU stay. Device exposure and infection status were used as time-varying variables. Logistic regression models for the day-by-day incidence of IAP, BSI, intubation, CVC, discharge from the ICU, and death in the ICU, were computed. Furthermore, using the specific probabilities of exposure and infection from the corresponding logistic models, for each day and patient, device exposure and infection status were simulated obtaining predicted values used for ICU classification as “best ICUs” and “other ICUs”. Finally, in order to obtain expected values from the model, simulation with standardization (WS) was performed.

The preventable number of IAP was calculated using the formula of Predicted cases minus Expected cases.

Likewise, a further evaluation of the preventable proportion was performed using data on compliance with the European bundle components. In particular, in this simulation analysis, ICU quality was selected using compliance with the IAP bundle components. Thus, the “best ICU” was the ICU with the highest percentage of patients with compliance to all five components of bundle. Finally, as mentioned before, predicted and expected values of IAP, considering the “best ICU” for bundle compliance were computed.

The SPSS software (IBM Corp. Released 2013. IBM SPSS Statistics for Windows, Version 22.0. Armonk, NY: IBM Corp.) was used to perform backward Logistic Regression analyses, and R, version 2.13.1. was used to perform Monte Carlo simulations.

## Results

During the fourth edition of the SPIN-UTI project, a total of 3,009 patients were enrolled by 26 ICUs. For the present analysis, 1,169 patients (38.8% of the total) and 9 ICUs, were excluded (see S1 Text for details). Thus, a total of 1,840 patients (of which 1,494 with intubation) and of 17 ICUs were included in the analysis and their characteristics are detailed in Table 1.

**Table 1:** Patients and Intensive Care Unit characteristics.

Variables*	
Patients	1,840
Females	764 (41.5)
Trauma	82 (4.5)
Impaired immunity	117 (6.4)
Administration of antibiotics within 48 hours of admission	1,092 (59.3)
Type of ICU admission - Medical - Scheduled surgery - Unscheduled surgery	847 (46.0) 473 (25.7) 520 (28.3)
Origin of patients - Other ward of this/other hospital - Other ICU - Community (home) - Long-term care facility	1,371 (74.5) 38 (2.1) 408 (22.2) 23 (1.3)
Median length of stay in ICU in days (IQR)	7 (4–14)
Median age in years (IQR)	70 (57–78)
Median SAPS II score at admission (IQR)	39 (28–52.8)
ICUs	17
ICU type - Mixed - Surgical	16 (94.1) 1 (5.9)
Median number of beds (IQR)	8 (5–11.5)
Median duration of intubation in days (IQR)	5 (2–12)
Median mortality in the ICU (IQR)	18 (15.4–32.6)
Median percentage of intubated patients at admissions (IQR)	89.4 (68.6–95.6)
ICU-acquired infections - Median IAP per 100 intubated patients (IQR) - Median IAP per 1,000 intubation-days (IQR)	11.7 (5.1–21.2) 11.8 (5.2–20.4)

* Categorical variables are reported as number (%).

Abbreviations: ICU, intensive care unit; IQR, interquartile range; SAPS, Simplified Acute Physiology Score; IAP, Intubation-Associated Pneumonia.

Table 2 shows the number of observed, predicted, and expected cases of IAP, the preventable number and proportion of IAP. Thus, a proportion of 44% of IAP was estimated to be preventable.

**Table 2:** Observed, predicted, and expected cases of IAP and preventable number and proportion of IAP.

	Best ICUs (n = 2)		Other ICUs (n = 15)			Preventable Number	Proportion of simulated mean of cases preventable (±SD)
	Observed	Mean Predicted	Observed	Mean Predicted	Mean Expected		
Number of IAP	3	4.55	225	181.9	95.2	86.7	0.44 (±0.06)
IAP per 100 patients with intubation	2.05 (3/146)	2.28 (4.55/200)	16.5 (225/1362)	11.1 (181.9/1639.3)	5.81 (95.2/1639.1)	5.29	0.50 (±0.06)
IAP per 1,000 Intubation-days	6.76 (3/444)	5.86 (4.55/776.35)	15.82 (225/14220)	12.64 (181.9/14389.95)	8.61 (95.2/11057.6)	4.03	0.30 (±0.08)

Abbreviations: ICU, intensive care unit; IAP, Intubation-Associated Pneumonia; SD, standard deviation.

**Fig 1:**
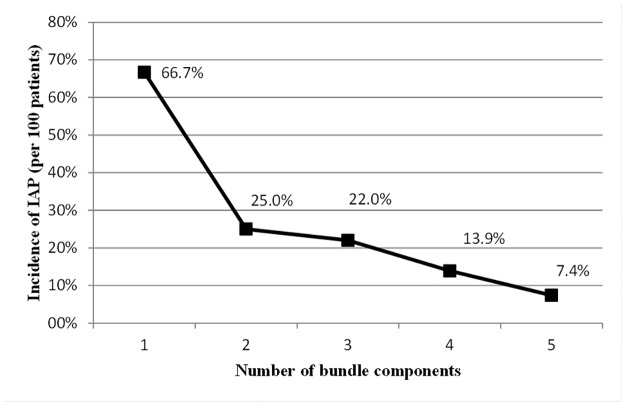
Correlation between incidences of IAP and number of bundle components performed. The correlation between incidences of IAP and the number of bundle components performed was assessed using linear regression and the Pearson correlation coefficient. A significant negative trend of IAP incidences was observed with increasing number of bundle components performed (p<0.001) and a strong negative correlation between these two factors was shown (r = -0.882; p = 0.048).

Data on bundle compliance were available for a total of 768 intubated patients admitted in the 15 participating ICUs. The results, described in great details elsewhere [9, 10], showed that the components of the European bundle are implemented at different levels in the participating ICUs. A high level of compliance with bundle practices was found except for the daily sedation vacation and weaning protocol. Overall compliance with all five practices included in the bundle was reported for 21.1% of the included patients [9, 10]. A significant negative trend of IAP incidences was observed with increasing number of bundle components performed (p<0.001) and a strong negative correlation between these two factors was shown (r = -0.882; p = 0.048) (Fig 1). Furthermore, the IAP rate was 21.5 per 1000 intubation-days, when only one component of the bundle was performed, decreasing up to 11.2 per 1000 intubation-days, when all the five bundle components were applied.

Finally, from the model-based simulation, considering compliance with the European bundle, if all ICUs had the same percentage of patients reporting compliance with all five components as the “best” ICU, a proportion of 40% of IAP was estimated to be preventable (Table 3).

**Table 3:** Observed, predicted, and expected cases of IAP and preventable number and proportion of IAP, considering compliance with the European bundle.

	Best ICU (n = 1)		Other ICUs (n = 9)			Preventable Number	Proportion of simulated mean of cases preventable (±SD)
	Observed	Mean predicted	Observed	Mean predicted	Mean expected		
Number of IAP	4	4.5	107	91	50.9	40.10	0.40 (±0.09)

Abbreviations: ICU, intensive care unit; IAP, Intubation-Associated Pneumonia; SD, standard deviation.

## Discussion

ICU-acquired pneumonia has been associated with clinically important outcomes, including duration of mechanical ventilation or intubation, length of ICU-stay and increased mortality rates and healthcare costs [16–18]. The prevention of this severe infection has been the focus of numerous studies in critically ill patients and remains a controversial issue [19]. Different factors associated with infection rates can be targeted in order to control their incidence [12]. Particularly, the management of intubation procedures has been identified as a potential target for infection control interventions and, as such, there is the need for implementation of strategic bundles in order to decrease the growing risk of ICU-acquired pneumonia [8].

Different estimates have been reported about the preventable proportion of HAIs under routine working conditions. Although 100% preventability may not be attainable, evidence-based infection control strategies could prevent a large proportion of infections [20]. In 1975, the Study on the Efficacy of Nosocomial Infection Control (SENIC) project estimated that 22% of cases of pneumonia were preventable with effective surveillance and control programs [21]. However, only a fraction of HAIs were actually being prevented, because many hospitals had not implemented recommended infection control measures [20, 22, 23]. The systematic review by Harbarth et al., [24] estimates that at least 20% of all HAIs are probably preventable and, depending on the setting, study design, baseline infection rates and type of infection, the reduction effect ranged from 10% to 70%. The European Centre for Disease prevention and Control reports that about 20–30% of HAIs are preventable by intensive hygiene and control programmes (http://ecdc.europa.eu/en/healthtopics/Healthcare-associated_infections/Pages/index.aspx). Furthermore, in United States hospitals, 55% of cases of ventilator-associated pneumonia may be preventable with current evidence-based strategies [20].

The study by Lambert et al., [12] on a large database from European surveillance networks, including the SPIN-UTI network, estimated that 52% of ventilator-associated pneumonia is preventable [12]. In our study, using a similar approach on prospectively collected patient-based data, allowing for case mix adjustment, we have estimated in the first model that a proportion of 44% of IAP was shown to be preventable.

Remarkably, in the second model-based simulation conducted in a subgroup of our population, where bundle compliance was added in the statistical model, a proportion of 40% of IAP was shown to be preventable, highlighting the important role of good clinical practices among the other factors that can be targeted by appropriate interventions of infection control. However, the small number of patients included in the care bundle compliance analysis, may represent a limit of the study and further and larger studies should be conducted.

Notably, in the SPIN-UTI network a low level of compliance with all five components of the European bundle has been reported [9, 10] confirming a percentage comparable with that reported at European level (21.1% versus 20% of patients) [14]. Nevertheless, collection of accurate data on compliance using process measures is particularly difficult and the bundle components themselves are still controversial.

A previous study has demonstrated a simultaneous increasing bundle compliance trend and a decreasing trend of ventilator-associated pneumonia rate [25]. Interestingly, in the present study, a significant negative trend of IAP incidence with increasing number of bundle components performed was observed. Furthermore, a strong negative correlation between these two factors was shown, suggesting that considerable improvements in infection control can be achieved with a higher degree of compliance with the components of the European bundle for IAP prevention.

In the present study, the IAP definition according to the HAI-ICU protocol [4] was used. However, it should be pointed out the subjective nature of the definition of IAP that includes clinical and radiological criteria, which suffer from subjectivity and variability, inherent in poor documentation in the medical record and chest radiograph technique and interpretation. Thus, this definition has a low kappa score because of lack in sensitivity and specificity [26–27].

The limitations of ventilator-associated pneumonia or IAP surveillance definitions could have implications for prevention. Recently, in order to come up with a more objective definition, the Centers for Disease Control and Prevention (CDC) developed a new algorithm to diagnose Ventilator-Associated Events (VAE) instead of ventilator-associated pneumonia or IAP [28].

Although implementation of single separate interventions might improve patient care, the simultaneous implementation of some simple measures included in a bundle has a greater likelihood of improving patient outcome [29]. However, the limit of the observational study design, that cannot definitively prove a causative relationship between bundle compliance and IAP risk, should be considered. In fact, the study does not account for different other factors that could be involved, as other specific preventive and infection control practices and measures that may have been implemented in the participating ICUs. Thus, this issue should be clarified in further studies with a randomized design. Additionally, in the present study, the impact of each bundle elements on IAP risk, in terms of preventable proportion, was not assessed. However, a recent study which evaluated the associations between individual and collective ventilator bundle components and VAE, reported that performing 4 measures together was significantly associated with lower risk for ventilator mortality, but not for VAE. Besides, single bundle components were both associated with negative outcomes (i.e. daily oral care with chlorhexidine and stress ulcer prophylaxis), with lower risk of VAE (i.e. spontaneous breathing trials) and lower hazard for ventilator mortality (i.e. sedative infusion interruptions) [30].

Furthermore, it has been pointed out that infections that seem to be most amenable to infection control measures are those that result from transmission between patients [31]. Thus, the proportion of ICU-acquired infections that are a consequence of nosocomial cross-transmission between patients should be considered when computing estimates of preventable proportion of infection in ICU [31, 32]. Active surveillance and epidemiological typing of associated clones has been implemented integrating the SPIN-UTI patient-based surveillance with a continuous laboratory-based monitoring programme [33]. The systematic use of this approach would provide the most comprehensive epidemiological *scenario* in order to better estimate the risk of infection to be reduced.

Education is not sufficiently effective in improving bundle compliance among nurses and ICU staff [34] and frequent and continuous recall of the necessity of the bundle and strict supervision of compliance are needed. A systematic review conducted in order to assess the effectiveness of different interventions to improve professional adherence to infection control guidelines on device-related infection rates, reports that interventions that may be worth further study are educational interventions involving more than one active element, repeatedly administered over time, and employing specialised personnel [35].

In conclusion, the g-formula controlled for time-varying factors, such as patients and ICU characteristics, is a valuable approach for estimating the preventable proportion of IAP and the impact of interventions, based entirely on an observed population in a real world setting [36]. Our results have shown a negative trend of IAP risk with increasing number of bundle components and suggest that a large proportion of IAP, i.e. 40%, can be prevented by improving adherence to good practices. However, the implementation of infection control strategies requires efforts in continuous healthcare worker’s education in order to obtain and maintain high levels of compliance [37,38]. Future interventions that aim to raise the compliance with bundle components in ICUs should be realised in order to improve patient’s outcomes.

## Supporting information

S1 Text Supplementary methods. Description of the statistical method.
(DOC)
