# Supplementary material for: Preventable proportion of intubation-associated pneumonia: Role of adherence to a care bundle
Source: PLoS One. 2017 Sep 6;12(9):e0181170. doi: 10.1371/journal.pone.0181170 (PMC5587225; doi:10.1371/journal.pone.0181170)
Supplement: S1 Text — Description of the statistical method. (DOC) [file pone.0181170.s001.doc]

# Supplementary methods

This document describes in details the statistical method adopted in the article "Preventable Proportion of Intubation-Associated Pneumonia: Role of Adherence to a Care Bundle Approach" by Agodi et al.

The aim of this analysis was to estimate the expected number of Intubation-Associated Pneumonia (IAP) and the incidence that would be realized if Intensive Care Units (ICUs) with high IAP incidence had the same incidence as reference ICUs after adjusting for patients and ICU characteristics.

## Overall preventable proportion (pp) evaluation - study population

The original database of the Italian Nosocomial Infections Surveillance in ICUs (SPIN-UTI) network includes 3,009 patients enrolled in 26 ICUs. The total number of IAP was 271.

The SPIN-UTI database was cleaned according to the following exclusion criteria. All records for patients from a given ICU were excluded from the analysis if data regarding patients characteristics and day-to-day exposure to invasive devices (i.e. intubation and Central Venous Catheter - CVC) were missing. ICUs which did not report any IAP and/or enrolled less than 20 patients (arbitrary threshold) were also excluded because of concerns regarding the possibility of inadequate infection detection and that data quality may be less good in these centres. Table 1 shows the number of patients, ICUs and IAP, excluded at each step, and the final numbers included in the analyses (Preventable Proportion, PP database).

Table 1. Number of patients, ICUs and IAP for overall PP

|  | Exclusion criteria | N of ICUs | N of Patients | N of IAP |
| --- | --- | --- | --- | --- |
| Original SPIN-UTI database |  | 26 | 3009 | 271 |
|  | No IAP | 3 | 730 | 0 |
|  | Patient missing data | 2 | 405 | 36 |
|  | Less than 20 patients | 4 | 34 | 7 |
| PP database |  | **17** | **1840** | **228** |

## The parametric g-formula for computing the preventable proportion of IAP

The model used in the present study has been previously adopted using a large European cohort study database [1]. The parametric g-formula, a generalization of standardization used to adjust for time-varying confounders affected by prior exposures [2], was used for computing the preventable proportion of IAP.

The prediction model was built through the following steps:

1. **Day-by-day database generation**
2. **Logistic regression analyses**
3. **Simulation without standardization (WOS)**
4. **Simulation standardization (WOS)**
5. From the PP database, by merging the patient, exposure and infection datasets, a day-by-day database was generated considering device exposure, IAP and bloodstream infection (BSI) status, for each patient and for each day of ICU stay. Device exposure and infection status were used as time-varying variables. In this way, the number of records per patient in the dataset equals the observed number of days in the ICU for that patient.

The first two days in the ICU per patient were not used to estimate the model parameters and the patient records from day 3 on in the ICU were used to generate the day-by-day database. ICU stays were cut at 100 days, limiting the effect of long ICU stays (the maximum observed ICU length was 131 days).

1. Patient baseline characteristics included in the logistic model were: age, sex, Simplified Acute Physiology Score II (SAPS II) at admission, origin of the patient, admission type, impaired immune status, antibiotic use at admission, and trauma. ICU characteristics were: ICU type, ICU mortality, proportion of intubated patients and of patients with a CVC.

For the categorical variables dummy codes were applied (using the last level as reference, when alphabetically sorted).

Backwards logistic regression models for the day-by-day incidence of IAP, BSI, intubation, CVC, discharge from the ICU, and death in the ICU, were computed. Significance level was set at 5%, removing the least significant main term at each step.

1. Based on the parameter estimates from the logistic models, the Monte Carlo simulation method was used to estimate the:

- number/fraction of IAP,
- number/fraction of IAP per 1000 intubation days, and
- number/fraction of IAP per 100 intubated patients.

This simulation, provided a set of crude “predicted” values which were adopted for ICU classification.

“Best ICUs” were defined those ICUs with the simulated IAP incidences below or equal to the 10^th^ percentile of the distribution; while “other ICUs” were considered those ICUs with higher IAP incidence, compared with the “best ICUs”.

4. Finally, in order to obtain the “expected” values from the model, simulation with standardization (WS) was performed. Through this step the predicted IAP rates obtained the “best ICUs” were applied to “other ICUs”.

The simulation was repeated 20 times and the results were averaged. In the validation of the regressive models used in the simulation, the deviation of the values predicted by the model from the observed values was between 10% and 22%.

Mean values ± standard deviation (SD) over the simulations were calculated.

The preventable number of IAP was calculated using the formula of “Predicted cases” minus “Expected cases”. As such, the formula is: N_IAP other_ICUs (WOS) - N_IAP other_ICUs (WS).

For each simulation, the PP of IAP is then given by the ratio of the preventable number of IAP divided by the total “predicted” number of IAP, obtained with the simulation WOS, and calculated using the formula: preventable number of IAP/ (N_IAP other_ICUs (WOS) + N_IAP best_ICUs (WOS).

# Bundle PP evaluation - study population

Likewise, a further evaluation of the preventable proportion was performed using data on compliance with the European bundle components, collected from a subgroup of ICUs, as described in the paper.

The Bundle database includes 768 patients enrolled in 15 ICUs and a total number of 120 IAP. The same exclusion criteria described above were adopted. Number of patients, ICUs and IAP excluded at each step and the final numbers included in the analyses are detailed in Table 2.

Table 2. Number of patients, ICUs and IAP for bundle PP

|  | Exclusion criteria | N of ICUs | N of Patients | N of IAP |
| --- | --- | --- | --- | --- |
| Original Bundle database |  | 15 | 768 | 120 |
|  | No IAP | 2 | 27 | 0 |
|  | Patient missing data | 1 | 49 | 6 |
|  | Less than 20 patients | 2 | 27 | 3 |
| PP Bundle database |  | **10** | **665** | **111** |

The prediction model adopted in this subgroup was built following the same scheme of the previous one.

The only exception was that, in the simulation WOS (step 3), ICU classification was estimated using compliance with the IAP bundle components. Thus, the “best ICU” was the ICU with the highest percentage of patients with compliance to all five components of bundle, and “other ICUs” were considered all others ICUs either with lower percentage of patients with compliance to all five components of bundle and with compliance to a lesser number of components.

Finally, as mentioned before, predicted and expected values of IAP, considering the “best ICU” for bundle compliance were computed using the same formula.

## Statistical software

The SPSS software (IBM Corp. Released 2013. IBM SPSS Statistics for Windows, Version 22.0. Armonk, NY: IBM Corp.) was used to perform backward logistic regression analyses, and R, version 2.13.1. was used to perform Monte Carlo simulations.

# References

1. Lambert ML, Silversmit G, Savey A, Palomar M, Hiesmayr M, Agodi A, et al. Preventable proportion of severe infections acquired in intensive care units: case-mix adjusted estimations from patient-based surveillance data. Infect Control Hosp Epidemiol 2014; 35:494-501.

2. Keil AP, Edwards JK, Richardson DR, Naimi AI, Cole SR. The parametric G-formula for time-to-event data: towards intuition with a worked example. Epidemiology. 2014; 25: 889–897.
